# Noncovalent Complexation of Amphotericin B with Poly(β-Amino Ester) Derivates for Treatment of *C. Neoformans* Infection

**DOI:** 10.3390/polym11020270

**Published:** 2019-02-05

**Authors:** Yang Yu, Li Peng, Guojian Liao, Zhangbao Chen, Chong Li

**Affiliations:** College of Pharmaceutical Sciences, Southwest University, Chongqing 400715, China; pengli201607@163.com (L.P.); gjliao@swu.edu.cn (G.L.); xiczb86@126.com (Z.C.)

**Keywords:** MPEG-PLA-PAE, polymer complex, enhanced stability, amphotericin B, *C. neoformans* infections, drug delivery

## Abstract

Our goal was to improve treatment outcomes for *C. neoformans* infection by designing nanocarriers that enhance drug-encapsulating capacity and stability. Thus, a noncovalent complex of methoxy poly(ethylene glycol)-poly(lactide)-poly(β-amino ester) (MPEG-PLA-PAE) and amphotericin B (AMB) was developed and characterized. The MPEG-PLA-PAE copolymer was synthesized by a Michael-type addition reaction; the copolymer was then used to prepare the AMB-loaded nanocomplex. AMB was in a highly aggregated state within complex cores. A high encapsulation efficiency (>90%) and stability of the AMB-loaded nanocomplex were obtained via electrostatic interaction between AMB and PAE blocks. This nanocomplex retained drug activity against *C. neoformans* in vitro. Compared with micellar AMB, the AMB nanocomplex was more efficient in terms of reducing *C. neoformans* burden in lungs, liver, and spleen, based on its improved biodistribution. The AMB/MPEG-PLA-PAE complex with enhanced drug-loading capacity and stability can serve as a platform for effective treatment of *C. neoformans* infection.

## 1. Introduction

*Cryptococcus neoformans* (*C. neoformans*) is a human fungal pathogen and a major cause of fungal meningitis in immunocompromised patients [1,2,3]. Amphotericin B (AMB) is a polyene macrolide antifungal agent and the preferred drug for the treatment of cryptococcal infection. Owing to its poor solubility in water, several AMB formulations are currently available, including a micellar form with sodium deoxycholate as a surfactant (Fungizone) and lipid-based formulations (such as an AMB lipid complex (ABLC) and a liposomal AMB (AmBisome)) [4,5]. However, serious toxicity or high treatment costs have limited the prevalence of AMB formulations to some extent [6]. Thus, much effort has been spent on developing less expensive delivery systems with reduced AMB toxicity.

Several polymer-based formulations have attracted considerable interest for delivering AMB [7,8]. In order to overcome its poor water solubility and stability, AMB can be entrapped into the hydrophobic core of nanoparticles using polymers such as PLA-*b*-PEG, (PEG)_3_-PLA, poly(ethylene oxide)-poly(N-hexyl-_L_-aspartamide), and gelatin [9,10,11,12]. However, application of these systems is still limited by a relatively low drug-loading capacity due to weak hydrophobic interactions between AMB and these polymers [9,10,11,12]. It was found by Mohamed-Ahmed et al. that poly(α-glutamic acid) could form a stable noncovalent complex with AMB at a loading content as high as 20%–50% [13]. The molecular self-assembly process of this complex relies on electrostatic interactions. In another study by Bastakoti et al., a cationic drug could be effectively incorporated into micelles of poly[styrene-*b*-sodium 2-(acrylamido)-2-methyl-1-propanesulfonate-*b*-ethylene oxide] (PS-*b*-PAMPS-*b*-PEO), and a high drug loading capacity was achieved of up to 1.2 g/g of polymer due to both the electrostatic attraction and hydrophobic interaction [14]. A similar approach was applied to incorporate cationic doxorubicin into micelles made of poly(styrene-*b*-acrylic acid-*b*-ethylene glycol) (PS-*b*-PAA-*b*-PEG) containing anionic PAA reaction sites [15]. Moreover, the ionic strength could affect particle size, zeta potential, drug-loading capacity, stability, drug release, and so on [16,17,18]. Therefore, the polyion complex on the strength of ionic interaction is a promising formulation for drug delivery.

Poly(β-amino ester) (PAE) is a type of pH-responsive biodegradable polymer that is insoluble in an alkaline environment, but soluble in acid via protonation of tertiary amino groups [19,20,21]. Our previous studies have shown that PAE blocks in copolymers could increase drug encapsulation efficiency and particle stabilization [22]. Thus, we hypothesized that the ionized PAE blocks could closely associate with AMB based on electrostatic interactions, which may form a stable water-soluble nanocomplex. With this aim, AMB-loaded nanocomplexes were prepared with MPEG-PLA-PAE copolymers, and various characteristics such as particle size, zeta potential, drug-entrapping efficiency, stability, and in vitro drug release were studied. Additionally, we investigated the in vivo circulation, biodistribution, and therapeutic efficacy of the nanocomplex in mice experimentally infected with *C. neoformans*.

## 2. Materials and Methods

### 2.1. Reagents and Materials

Amphotericin B (AMB) and poly(ethylene glycol) methyl ether (MPEG_2000_) were purchased from Sigma-Aldrich (St. Louis, MI, USA). Acryloyl chloride, triethylamine, 4,4-trimethylene dipiperidine (TDP), tin(II)2-ethylhexanoate (Sn(Oct)_2_), and 1,10-decanediol diacrylate (DDD) were bought from TCI Chemicals (Tokyo, Japan). Amphotericin B for injection, produced by North China Pharmaceutical Group Corporation (Hebei, China), is a complex of amphotericin B and sodium desoxycholate. This injection solution was used as a control and named as “AMB″.

*C. neoformans* (H99) was incubated in yeast extract peptone dextrose (YPD) medium at 30 °C. Six-week-old Kunming mice (KM) weighing 20 ± 2 g and Sprague-Dawley (SD) rats weighing 200 ± 20 g used in this study were purchased from Chongqing Institute of Chinese Medicine (Chongqing, China). The animals were housed in laboratory animal environment of SPF. All animal experiments were carried under the guidelines of the Ethical Review Committee of experimental animals at Southwest University of China (Approval #: SWU2017/7).

### 2.2. Synthesis of MPEG-PLA-PAE Copolymers 

**Figure 1:**
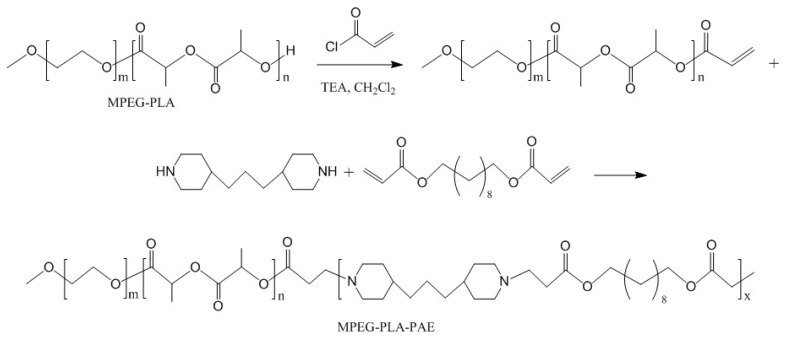
Synthesis scheme of MPEG-PLA-PAE.

The entire synthesis route for MPEG-PLA-PAE is illustrated in Figure 1. Firstly, MPEG-PLA copolymers were synthesized by a ring-opening polymerization of _D, L_-lactide using Sn(Oct)_2_ as a catalyst and MPEG_2000_ as a macroinitiator. MPEG-PLA acrylate was synthesized as follows: MPEG-PLA (1 eq.) and triethylamine (2 eq.) were dissolved in anhydrous dichloromethane, followed by dropwise addition of acryloyl chloride (1.5 eq.) at 0 °C. After a 24-h reaction at room temperature, the solution was washed with 1 M HCl solution, brine, and water. The organic phase was dried over Na_2_SO_4_ and concentrated in vacuo, followed by precipitation with diethyl ether. Finally, the MPEG-PLA-PAE was synthesized by Michael addition of TDP (1.1 eq.) to MPEG-PLA acrylate (0.1 eq.) and DDD (1 eq.). The mixture was dissolved in anhydrous chloroform and then stirred at 50 °C for 48 h under nitrogen; the final product was precipitated in cold diethyl ether and vacuum dried at room temperature.

The polymer structure was characterized by ^1^H NMR spectroscopy (Bruker AVANCE III HD 400) using CDCl_3_ as the solvent. The molecular weight of copolymers was determined by gel permeation chromatography (GPC) with two Styragel columns (Shodex KF-802.5 and KF-803L). The molecular weights of the polymers were calibrated against PEG standards (Waters) with the molecular weight ranging from 420–22,100 using tetrahydrofuran as a mobile phase [19].

### 2.3. Preparation and Characterization of AMB-Loaded Nanocomplexes

The AMB-encapsulated complex was produced by an emulsion solvent evaporation method. Briefly, 20 mg of MPEG-PLA-PAE were dissolved in 3 mL of acidic water (pH 2–3), while 2 mg of AMB were dissolved in dichloromethane-methanol-0.05 M HCl (10:4:1) solvent. These two solutions were mixed by ultrasonication for 1 min under cooling, followed by evaporation of organic solvents under reduced pressure at 40 °C. The obtained solution was dialyzed (MWCO 8000–14,000, Millipore Corporation, MA, USA) against deionized water for 3 h and then filtered with a microfiltration membrane (0.45 μm). 

The AMB/MPEG-PLA micelles were prepared using a thin-film hydration method. In brief, 20 mg of MPEG-PLA were dissolved in 2 mL of chloroform and then mixed with 2 mg of AmB in methanol solution. The organic solvents were evaporated under high vacuum at 50 °C to produce a thin drug/polymer film, followed by hydration with 3 mL of pure water. This solution was sonicated for 1 min and then filtered with a microfiltration membrane (0.45 μm). 

Particle size and zeta potential of the complex were measured using dynamic light scattering (DLS, Malvern Nano ZS ZEN3600, Malvern Instruments Ltd., Worcestershire, UK) at 25 °C. The morphology of the complex was observed under transmission electron microscope (TEM, JEM-1230, JEOL Ltd., Tokyo, Japan) operating at an 80-kV accelerating voltage. X-ray diffraction (XRD) spectra of free AMB, copolymers, and AMB-loaded complex were determined using an X-ray diffractometer (Panalytical X’Pert^3^ Powder, PANalytical, Almelo, The Netherlands).

The aggregation state of AMB was determined by scanning the UV absorbance of AMB in the range of 300–450 nm using a UV-visible spectrophotometer. AMB gives four absorption peaks. The aggregation ratio was calculated as the ratio of Peak I (315–318 nm) to Peak IV (409 nm). 

### 2.4. HPLC Analysis for the Quantification of AMB 

In order to evaluate drug entrapment efficiency, free AMB was separated from the complex on a Sephadex G-25 column. High performance liquid chromatography (HPLC) was used for drug analysis, which was performed with a C18 column (Diamonsil C18, 5 μm, 150 × 4.6 mm) (mobile phase: acetonitrile-0.02 M EDTA (pH 4.5) (45:55, *v*/*v*), flow rate: 1.0 mL/min and UV detection at 386 nm [23]). 

Freeze-dried complex (or micelles) was used to measure the drug encapsulation efficiency, which was defined by the ratio of measured and initial amount of AMB encapsulated in complex (or micelles). The calculation equation is as follows: encapsulation efficiency (%) = actual amount of AMB in complex (or micelles)/initial amount of AMB in complex (or micelles) × 100.

The storage stability of the AMB-loaded complex was evaluated as follows: freshly-prepared complex solution was stored for seven days at 4 °C, and the drug content was determined at 0, 1, 2, 3, 5, and 7 days by HPLC. To examine the stability of the complex under physiological conditions, a solution of AMB-loaded complex was added to murine plasma (100 μg/mL of AMB) and incubated at 37 °C for 24 h. Aliquots were removed at 0, 1, 3, 6, and 24 h followed by analysis with HPLC. AMB retention was achieved from the weight ratio between the drug retained within complex and that added at 0 h. 

The in vitro release of AMB from each formulation was carried out by a dialysis membrane method. Briefly, 1 mL of particle solution (10 μg/mL) was transferred into a dialysis bag (MWCO 8000–14,000), which was then placed into 10 mL of phosphate buffer (containing 0.1% w/v of sodium deoxycholate, pH 7.4) and incubated at 37 °C with gentle agitation (100 rpm). At specified time intervals (1, 2, 4, 8, 12, 24, and 48 h), the release medium was completely replaced with fresh buffer, and the amount of AMB released in medium was estimated by HPLC.

### 2.5. Activity against C. Neoformans Cells In Vitro

Minimal inhibitory concentrations (MICs) for each AMB formulation were determined by the classical microdilution broth method. Briefly, *C. neoformans* cells grown in YPD liquid medium (1 × 10^3^ cells/mL) were transferred to wells containing varying concentrations of AMB in different formulations (16–0.06 μg/mL) in a 96-well plate. The plate was then incubated at 30 °C for 48 h, and the optical density of wells at 600 nm was measured to monitor cell growth. The lowest concentration that completely inhibited growth of the fungal cells was defined as MICs. The MICs were the average of measurements of three independent assays.

### 2.6. Pharmacokinetic and Biodistribution Studies

The pharmacokinetics of AMB-loaded injection solution, micelles, and complex were determined in SD rats. Each AMB formulation was administered at a dose of 1 mg AMB/kg body weight via intravenous (i.v.) route. Blood samples from rats were collected in heparinized tubes by retro-orbital puncture at each time point (5, 10, 15, and 30 min; 1, 2, 4, 8, 12, and 24 h). 

For biodistribution studies, each AMB formulation was injected via the tail vein at a dose of 1 mg AMB/kg body weight in Kunming mice. These mice were then sacrificed by ethyl ether inhalation at 1, 2, 6, 12, and 24 h; various tissues (lung, liver, spleen, heart, and kidney) were collected at each time point.

Samples were processed for AMB analysis according to a published procedure [18]. Blood samples were centrifuged at 3000 rpm for separation of plasma. For tissue processing, tissue samples (100 mg) were homogenized with 300 μL of saline in an ice bath. Then, 10 μL of internal standard solutions (1-amino-4-nitro naphthalene, 10 μg/mL) in methanol were spiked to 90 μL of plasma or tissue homogenization and vortexed for 2 min for uniform mixing, followed by the addition of 100 μL of DMSO and 200 μL of methanol. The mixtures were vortexed for 2 min followed by centrifugation (10,000 rpm, 4 °C, and 10 min), and the supernatant was collected for HPLC analysis. The main pharmacokinetic parameters were calculated by DAS software (Version 3.2.1). 

### 2.7. In Vivo Antifungal Activity of the AMB Nanocomplex

Mice were infected with 1 × 10^7^ *C. neoformans* via tail vein injection on Day 0. Then, each of the AMB formulations were administered via tail vein injection on Days 1, 2, and 3 at a dose of 1 or 3 mg AMB/kg body weight. Randomly-chosen control mice were given i.v. normal saline. After treatment, representative mice from each group were sacrificed on Days 4, 6, and 10. Tissue samples (lung, liver, spleen, brain, and kidney) from these mice were aseptically removed, weighed, and homogenized in sterile saline in a ratio of 1 g organ to 3 mL of saline. Homogenates were serially diluted (ten-fold) from 1:10–1:1000 with sterile saline, and 50 μL of each dilution were spread on YPD medium; fungal colonies were counted after 48 h of incubation at 30 °C.

To evaluate the potential toxicity of AMB to the liver and kidney, serum biochemical evaluations were performed on SD rats following injection of each AMB formulation every day for a week. Blood samples were withdrawn from the retro-orbital plexus, and plasma was separated by centrifugation at 3000 rpm for 15 min, which was then analyzed for biochemical parameters including alanine aminotransferase (ALT), aspartate aminotransferase (AST), creatinine (CREA), and urea (UREA).

## 3. Results and Discussion

### 3.1. Synthesis and Characterization of MPEG-PLA-PAE Copolymers

**Figure 2:**
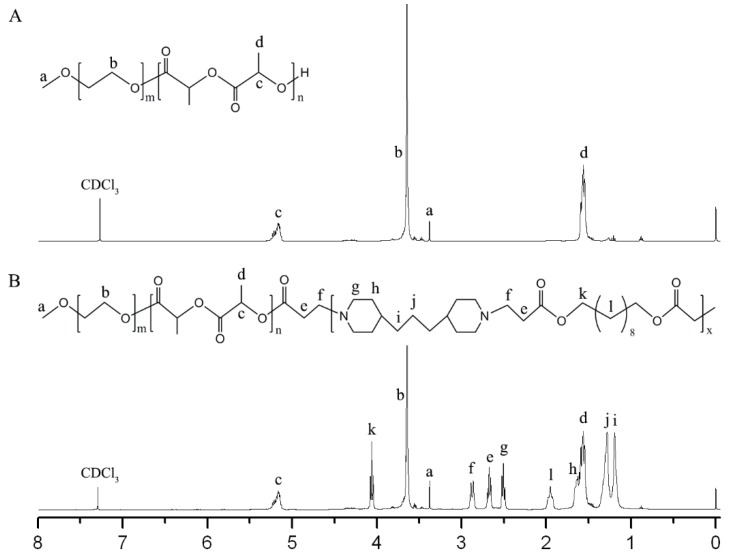
^1^H NMR spectra of (**A**) MPEG-PLA and (**B**) MPEG-PLA-PAE. CDCl_3_ was used as the solvent.

Firstly, MPEG-PLA was synthesized by ring-opening polymerization. As shown in Figure 2A, the integral values of peaks at 5.17 ppm (–CH of LA) and 3.65 ppm (–CH2 of EG) were used to determine the weight ratio of LA/EG (0.96) and the corresponding molecular weight of MPEG-PLA was 3920. From GPC analysis, the average molecular weight (Mn) and polydispersity index (Đ) were determined to be 4062 and 1.12, respectively. Then, MPEG-PLA-PAE was synthesized by Michael-type polymerization. As shown in Figure 2B, the characteristic signals of PLA were at 1.57 and 5.17 ppm, whereas those of MPEG were at 3.65 and 3.38 ppm. Peaks at 2.51, 1.63, 1.28, and 1.19 ppm were assigned to the TDP unit, whereas peaks at 4.07 and 1.95 ppm were assigned to the DDD unit. New peaks at 2.87 and 2.67 ppm confirmed the synthesis of copolymers. Almost no change in weight ratio of LA/EG was observed from integral values. From GPC measurement, the Mn and Đ of copolymers were determined to be 10,736 and 1.29, respectively.

### 3.2. Characterization of AMB-Loaded Nanocomplexes

An emulsion-solvent evaporation method was used to prepare the AMB/MPEG-PLA-PAE complex. To increase the water solubility of MPEG-PLA-PAE, a stoichiometric amount of 0.05 M HCl was added to deprotonate the tertiary amino groups of PAE. Meanwhile, AMB was dissolved in the dichloromethane-methanol-0.05 M HCl (10:4:1) solvent. During the mixing process, we hypothesized that the ionized PAE blocks would interact closely with AMB to induce complex formation.

AMB/MPEG-PLA micelles were relatively easy to fabricate by a thin-film hydration method rather than an emulsion-solvent evaporation method, with an encapsulation efficiency of 71.2% and 9.1%, respectively. The attachment of PAE chains to MPEG-PLA appears to promote emulsion formation in dichloromethane/water, generating a highly active drug encapsulation.

At the same feed, with a weight ratio of 2:20 of the drug:polymer, the drug encapsulation efficiency of AMB/MPEG-PLA-PAE (95.6%) was obviously higher than that of AMB/MPEG-PLA, indicating that the PAE segments in polymers could enhance the drug-loading capacity. The solubility of AMB (<1 μg/mL) was enhanced by approximately three orders of magnitude in the MPEG-PLA-PAE complex to 1.0–2.0 mg/mL (1000–2000-fold). However, it was noted that the drug encapsulation ability (70.5%) was obviously suppressed when using unionized MPEG-PLA-PAE. 

**Figure 3:**
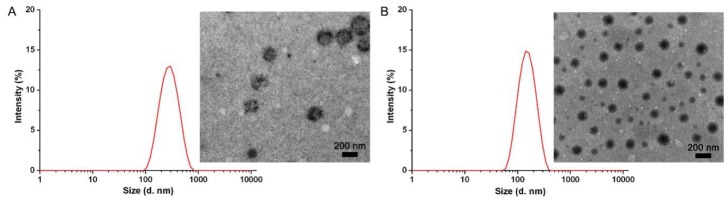
Size distribution and TEM images of (**A**) AMB/MPEG-PLA and (**B**) AMB/MPEG-PLA-PAE. Intensity (%) was achieved from the scattering intensity ratio between the particles and all species in the sample.

As shown in Table 1, the average size and zeta potential of AMB/MPEG-PLA were 205.7 ± 3.5 nm and 10.7 ± 0.8 mV, respectively. Due to the potential interactions between AMB and PAE segments, the size of the AMB/MPEG-PLA-PAE was generally smaller (by about 30%) than that of AMB/MPEG-PLA, and its zeta potential values increased to 35.1 ± 1.2 mV. The TEM images also show the AMB/MPEG-PLA-PAE to be compact, close to a spherical in shape, and smaller in size (Figure 3). Note that the surface charge of MPEG-PLA and MPEG-PLA-PAE particles without drug loading was 2.8 ± 0.2 and 27.0 ± 0.6 mV, respectively. Based on the results from other research [14,15], we hypothesized that both the ionic interaction and hydrophobic interaction contributed to the binding of AMB to MPEG-PLA-PAE, which was confirmed by changes of the zeta-potential and drug-encapsulation efficiency.

**Figure 4:**
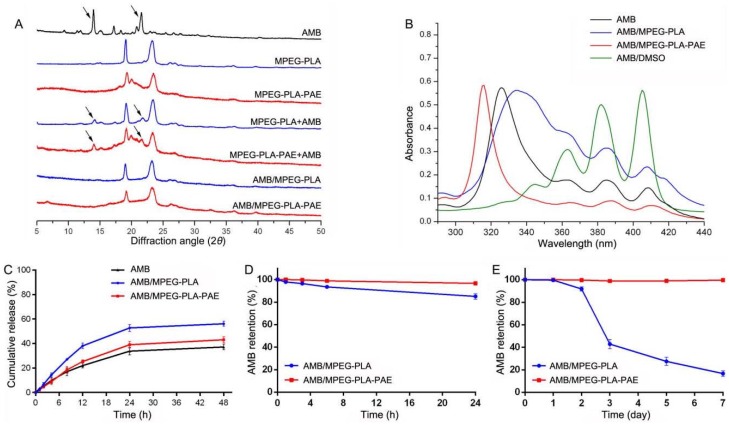
(**A**) XRD analysis of AMB, MPEG-PLA, MPEG-PLA-PAE, MPEG-PLA + AMB mixture, MPEG-PLA-PAE + AMB mixture, AMB/MPEG-PLA micelles, and AMB/MPEG-PLA-PAE complex. (**B**) Absorption spectra of AMB in injection solution, micelles, complex, and DMSO solution. (**C**) Release profile of AMB from each formulation. Cumulative release (%) was achieved from the weight ratio between the released drug and the total loaded drug. (**D**) Plasma stability and (**E**) storage stability of AMB/MPEG-PLA and AMB/MPEG-PLA-PAE. AMB retention (%) was achieved from the weight ratio between the drug retained within complex (or micelles) and that added at 0 h. All data are represented as the mean ± SD (*n* = 3).

XRD analysis was performed to examine the crystalline properties of AMB encapsulated in particles (Figure 4A). The characteristic XRD peaks of AMB disappeared in the spectrum of AMB/MPEG-PLA-PAE, implying that AMB was loaded into the nanocomplex in an amorphous state. Similar results were observed for the AMB/MPEG-PLA micelles.

It is reported that Fungizone shows peaks typically at 328, 365, 384, and 408 nm, whereas AmBisome shows a blue shift in the peak at 328 nm [11]. In this study, the AMB solution had an absorption spectrum similar to that of Fungizone, whereas the AMB/MPEG-PLA-PAE complex displayed a hypsochromic shift similar to AmBisome [10]. As shown in Figure 4B, the AMB/MPEG-PLA had a maximum absorption at 334 nm, and the other three peaks were more intense. AMB is monomeric in organic solvents (e.g., DMSO), but readily aggregates in an aqueous solution [24]. The monomeric form of AMB has a maximum absorption at 400–420 nm (IV), whereas aggregates display absorption at 300–350 nm (I). The ratio I:IV can be taken as a measure of the relative aggregation state of AMB. This ratio was quite low (<0.3) for monomeric AMB (AMB/DMSO) and was as high as 8.5 for highly-aggregated species (AMB/MPEG-PLA-PAE). It is known that AMB is highly aggregated in AmBisome, mostly due to the close association of AMB to the lipids [25]. Therefore, from the aggregated state of AMB in the hydrophobic core of the complex, we inferred that AMB might have a high affinity to PAE. It is known that the aggregation state of AMB may affect its toxicity, antimicrobial activity, and stability [26,27,28].

A release study was carried out with three formulations using 10-μg AMB equivalents by the dialysis method. AMB was released from each formulation in a biphasic profile with a fast release rate in the first 24 h, followed by a slow release (Figure 4C). The release rate of AMB from AMB/MPEG-PLA-PAE was obviously lower than that of AMB/MPEG-PLA, confirming the presence of good affinity between AMB and PAE segments. The total release of AMB from complexes and micelles was 43% and 56%, respectively.

Serum and storage stability for encapsulated AMB have been studied. As seen in Figure 4D, the AMB-entrapped complex formation showed better stability in serum than in its micellar formation. Maintaining a complex system during blood circulation may reduce AMB-induced toxicity [29,30]. In addition, the AMB/MPEG-PLA-PAE complex was stable for seven days at 4 °C with 2% AMB released into water. However, only 43% and 17% AMB remained intact in MPEG-PLA micelles after 3 and 7 days of incubation, respectively (Figure 4E). Similar findings have been reported in our previous study [31].

### 3.3. Activity against C. neoformans Cells In Vitro

As shown in Table 1, all AMB formulations exerted obvious antifungal activity against *C. neoformans* cells. The MIC value of the AMB nanocomplex was a little higher than that of the other two AMB formulations. It was thought that both AMB and its micellar system would rapidly dissociate into free AMB, which in turn would self-assemble into oligomers and thereby result in cytotoxicity [32,33]. In contrast, PAE blocks may suppress the formation of oligomers in the AMB complex due to the affinity between PAE and AMB. In addition, blank MPEG-PLA-PAE and MPEG-PLA solutions exhibited no antifungal activity against *C. neoformans* cells (MICs >200 μg/mL).

### 3.4. Pharmacokinetic and Biodistribution Studies

**Figure 5:**
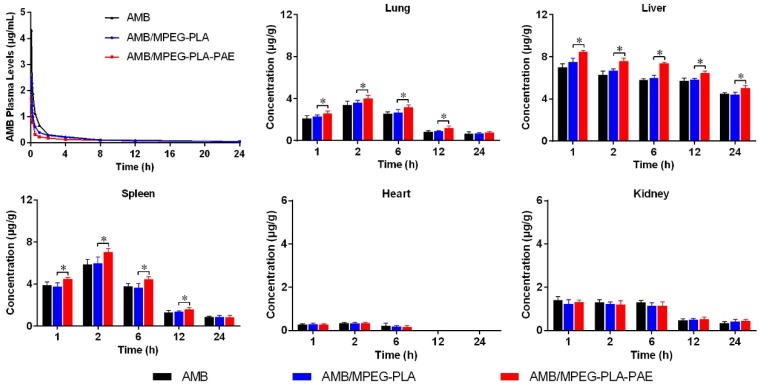
Plasma concentration-time curves and biodistribution after intravenous administration of AMB-loaded injection solution, micelles, and complex. Six rats from each group were used for pharmacokinetic studies at a dose of 1 mg AMB/kg body weight via intravenous route. For biodistribution studies, three mice at each time point were sacrificed after intravenous administration of each formulation of a dose of 1 mg AMB/kg body weight. Significant differences in tissue drug content (lung, liver, and spleen) between the AMB/MPEG-PLA and AMB/MPEG-PLA-PAE group are indicated with * *p* < 0.05 (data represented as the mean ± SD).

The plasma concentration-time profiles showed a rapid initial distributive phase within 2 h, followed by a slower elimination phase until 24 h (Figure 5). At each time point, the drug concentration of the AMB/MPEG-PLA-PAE group was lower than that of the AMB/MPEG-PLA and AMB group. The pharmacokinetic parameters were calculated by a non-compartment model. Both AMB-loaded complexes and micelles provided lower AUC_0–t_ values and higher total clearance (CL_z_) values (Table 2) than the AMB solution. The AMB-loaded complex had the fastest clearance rate from blood among all AMB formulations.

It is known that positively- and negatively-charged particles tend to bind with numerous serum proteins in the blood stream. After protein coating, nanoparticles are more easily taken up by macrophages and thereby exhibit reduced circulation time [34,35,36]. Therefore, the high positive charge of AMB/MPEG-PLA-PAE (35.1 ± 1.2 mV) resulted in its rapid blood clearance. 

The tissue distribution of the three different AMB formulations is illustrated in Figure 5. After i.v. administration of the drug, the heart showed detectable drug levels just within 6 h, whereas drug levels in other different organs could be detectable within 24 h. Compared to AMB and AMB/MPEG-PLA, AMB/MPEG-PLA-PAE had a higher degree of accumulation in the liver and spleen (*p* < 0.05), which may be attributed to its high positive charge. The exposure of positive charge would facilitate rapid uptake of particles into macrophages in the reticuloendothelial system located in the liver and spleen. In the lung, there was also a higher accumulation of the positive complex (*p* < 0.05). Similar findings have been reported in other studies; for example, one study reported that cationic lipid-based DNA complexes appeared mainly in the lung, liver, and spleen when delivered intravenously [37]. A possible explanation for this phenomenon was that positively-charged particles might form aggregates with blood cells by electrostatic interaction followed by entrapment in lungs. In the heart and kidney, there were no differences in the distribution of each formulation. 

### 3.5. In Vivo Antifungal Activity of AMB Nanocomplexes

**Figure 6:**
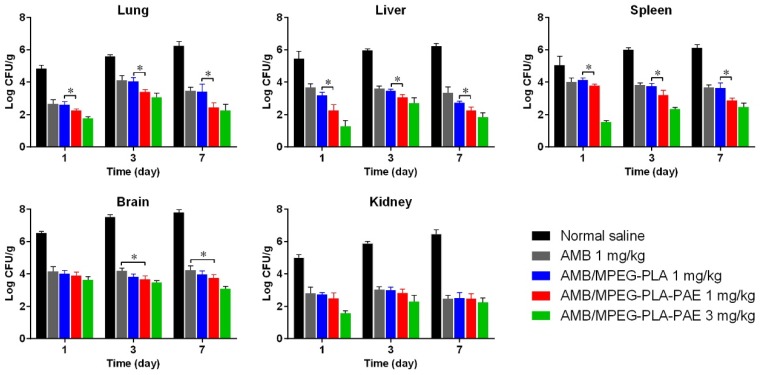
Total burden (lung, liver, spleen, brain, and brain) of mice infected with *C. neoformans* and treated with either normal saline or the various AMB formulations at 1 or 3 mg/kg (data represented as the mean ± SD; n = 6 for the different treatment groups). Significant differences in fungal tissue burden (lung, liver, and spleen) between the AMB/MPEG-PLA and AMB/MPEG-PLA-PAE group are indicated with * *p* < 0.05. In addition, fungal burden in brain tissue was significantly (*p* < 0.05) different between the AMB and AMB/MPEG-PLA-PAE group.

Based on the results from in vitro assays, the AMB-loaded nanocomplex was used for further evaluation in the mice model infected with *C. neoformans*. As shown in Figure 6, viable *C. neoformans* was recovered from liver, spleen, lungs, kidneys, and brain. The amount of *C. neoformans* in tissues increased rapidly with days, but decreased significantly after treatment. No viable colonies of *C. neoformans* were detectable in the heart of infected mice in each group. As expected, AMB/MPEG-PLA-PAE reduced the *C. neoformans* burden in lungs, liver, and spleen more efficiently than AMB/MPEG-PLA (*p* < 0.05), which could be due to its distinct surface properties and tissue-specific distribution. Moreover, the AMB complex at 3 mg/kg could further reduce fungal burden in mice as compared to that at 1 mg/kg. At the same dose (3 mg/kg), AMB and AMB/MPEG-PLA, could cause acute toxic reactions leading to the death of mice after i.v. administration. It was reported that the maximum tolerated dose of Fungizone was 2 mg/kg, and the 50% lethal dose was 2.5 mg/kg. Thus, it appears that the AMB nanocomplex was able to reduce AMB toxicity to some extent.

**Figure 7:**
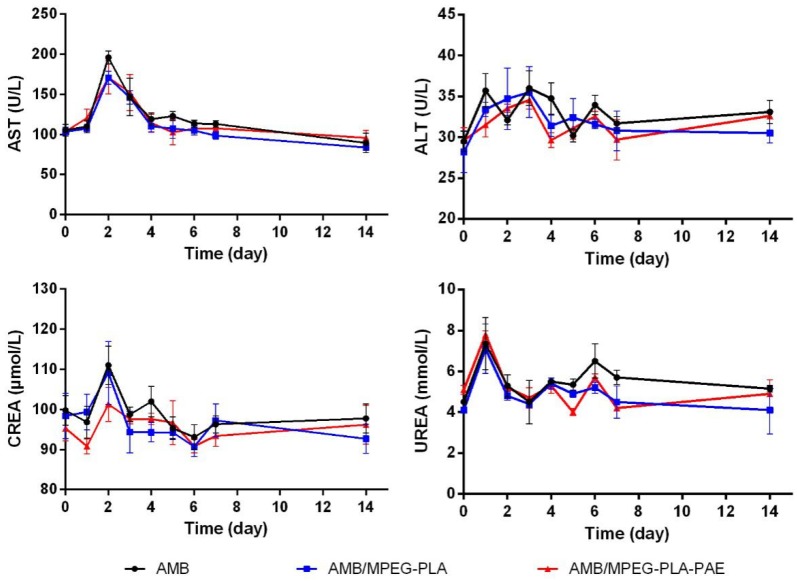
Levels of serum biochemical indicators (AST, ALT, CREA, and UREA) in rats during one-week treatment of various AMB formulations. Six rats each group were used for serum biochemical evaluations at a dose of 1 mg AMB/kg body weight by intravenous administration every day for a week (data represented as the mean ± SD).

The potential toxicity of AMB to liver and kidneys was evaluated by measuring the levels of serum biochemical indicators in rats during the one-week treatment period. As seen in Figure 7, there was no significant difference in the serum biochemical indicators (AST, ALT, CREA, and UREA) among study groups during the experimental period, indicating that no obvious side effects on hepatic and renal function were observed after injection of the AMB-entrapped nanocomplex.

## 4. Conclusions

In the current study, we developed a noncovalent complex for AMB delivery based on MPEG-PLA-PAE copolymers. Due to the electrostatic interactions between AMB and PAE blocks, entrapped AMB in the nanocomplex showed dramatically increased water solubility, high loading capacity, and enhanced stability. In vivo antifungal activity tested against *C. neoformans* in mice was found to improve the therapeutic benefits of AMB significantly when loaded within the nanocomplex. Moreover, no obvious side effects on liver and kidneys were observed after treatment with the AMB nanocomplex. Overall, our results indicate that we have designed a suitable nanocarrier for efficient delivery of AMB in the treatment of *C. neoformans* infection.

## Figures and Tables

**Table 1:** Characterization of each formulation.

Sample	Encapsulation Efficiency (%)	I/IV	Size (nm)	Polydispersity Index (PDI)	Zeta Potential (mV)	MIC (μg/mL)
AMB	-	4.0	-	-	-	0.5
MPEG-PLA	-	-	20.4 ± 1.4	1.08 ± 0.02	2.8 ± 0.2	-
MPEG-PLA-PAE	-	-	106.4 ± 2.9	1.13 ± 0.02	27.0 ± 0.6	-
AMB/MPEG-PLA	71.2 ± 2.5	2.6	205.7 ± 3.5	1.12 ± 0.02	10.7 ± 0.8	0.5
AMB/MPEG-PLA-PAE	95.6 ± 1.2	8.5	115.8 ± 2.8	1.22 ± 0.03	35.1 ± 1.2	0.5–1
AMB/DMSO	-	0.3	-	-	-	-

**Table 2:** Pharmacokinetic parameters of various AMB formulations (1 mg/kg).

Pharmacokinetic Parameter	Value for Formulation (mean ± SD)		
	AMB	AMB/MPEG-PLA	AMB/MPEG-PLA-PAE
AUC_0–t_ (mg·h/L)	3.47 ± 0.18	2.42 ± 0.19	1.59± 0.07
AUMC_0–t_ (mg·h^2^/L)	6.29 ± 0.51	5.46 ± 0.51	3.85 ± 0.18
*t*_1/2z_ (h)	4.02 ± 0.03	3.59 ± 0.06	3.57 ± 0.07
*V*_z_ (L/kg)	1.41 ± 0.07	1.76 ± 0.12	2.74 ± 0.09
CL_z_ (L/h/kg)	0.24 ± 0.01	0.34 ± 0.03	0.53 ± 0.03
